# Turning dyneins off bends cilia

**DOI:** 10.1002/cm.21483

**Published:** 2018-09-16

**Authors:** Stephen M. King

**Affiliations:** ^1^ Department of Molecular Biology and Biophysics University of Connecticut Health Center Farmington Connecticut

**Keywords:** axoneme, *Chlamydomonas*, cilia, dynein, flagella, microtubule, sea urchin

## Abstract

Ciliary and flagellar motility is caused by the ensemble action of inner and outer dynein arm motors acting on axonemal doublet microtubules. The switch point or switching hypothesis, for which much experimental and computational evidence exists, requires that dyneins on only one side of the axoneme are actively working during bending, and that this active motor region propagate along the axonemal length. Generation of a reverse bend results from switching active sliding to the opposite side of the axoneme. However, the mechanochemical states of individual dynein arms within both straight and curved regions and how these change during beating has until now eluded experimental observation. Recently, Lin and Nicastro used high‐resolution cryo‐electron tomography to determine the power stroke state of dyneins along flagella of sea urchin sperm that were rapidly frozen while actively beating. The results reveal that axonemal dyneins are generally in a pre‐power stroke conformation that is thought to yield a force‐balanced state in straight regions; inhibition of this conformational state and microtubule release on specific doublets may then lead to a force imbalance across the axoneme allowing for microtubule sliding and consequently the initiation and formation of a ciliary bend. Propagation of this inhibitory signal from base‐to‐tip and switching the microtubule doublet subsets that are inhibited is proposed to result in oscillatory motion.

## 1 INTRODUCTION

The rhythmic beating of cilia and flagella (here the term cilia is mainly used throughout) is driven by the coordinated action of multiple dynein ATPase motors generating inter‐doublet microtubule sliding (Satir, 1968; Summers & Gibbons, 1971). For microtubule sliding to be converted into a ciliary bend, dynein activity must be tightly controlled so that motors on only one side of the axoneme are active; this region of active motors must then propagate along the ciliary structure. To generate a bend in the reverse direction, the region of active sliding must switch to the opposite side of the axoneme. Numerous experimental and computational results support this “switch point” hypothesis which predicts that within a ciliary bend, dyneins on one side of the axoneme will be in a different functional state to those on the opposite side (Shingyoji et al., 1977; Wais‐Steider & Satir, 1979; Brokaw, 1991, 2009; Bayly & Dutcher, 2016; Sartori et al., 2016; King & Sale, 2018; Shingyogi, 2018); this switching must occur at a rate consistent with the ciliary beat frequency which in some organisms can approach 100 Hz. Although this model does not dictate particular dynein state transitions within straight or curved axonemal regions, there has been a general presumption that it likely involves the activation of specific dynein subsets as the bend travels from the ciliary base to the tip. However, until recently what those functional states might be and how they change with time had eluded experimental observation.

Now in a recent report, Lin and Nicastro (2018) use cryo‐electron tomography (cryo‐ET) to differentiate between pre‐ and post‐power stroke conformations of dynein motors along the length of sea urchin sperm flagella that were actively beating at the instant they were frozen, thereby preserving their functional state. This was achieved by using sub‐tomogram averaging to obtain a 30 Å‐resolution class average structure which was then used as a template to differentiate between the pre‐ and post‐power stroke conformations in non‐averaged structures. The key result to come from this study is that within unbent regions of the cilium, dyneins on both sides of the axoneme are in the primed microtubule‐attached pre‐power stroke conformation and thus there is a balance of forces around the structure. However, within bends dyneins on one side of the axoneme are in an “inhibited” or microtubule‐released state, leading to a force imbalance which allows opposing dyneins to undergo a power stroke causing sliding and thus bending. Propagation of this inhibitory state along the axoneme thereby leads to axonemal beating. This “switch inhibition” model allows for a robust all‐or‐none response, provides new insight into the mechanisms of oscillatory ciliary beating, and highlights essential issues that need be addressed to understand the underlying signal propagation mechanism.

## 2 DYNEIN STRUCTURE AND ARRANGEMENT IN THE AXONEME

**Figure 1:**
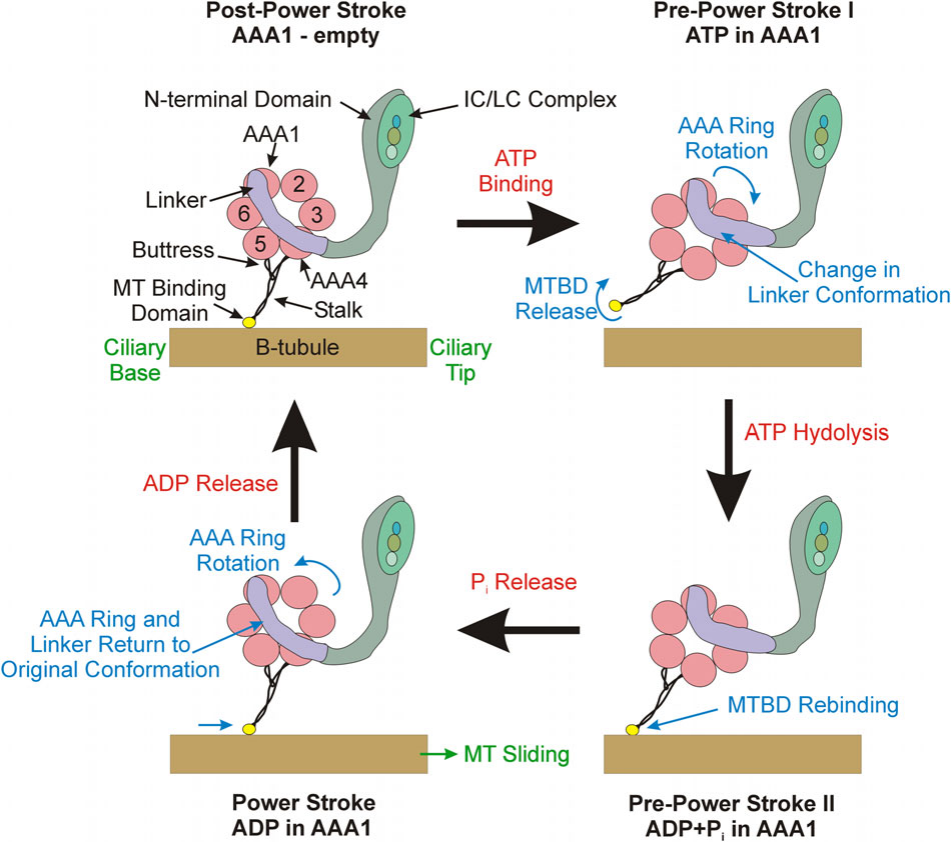
Dynein organization and power stroke structural transitions. This diagram illustrates the four major transitions that occur during the dynein mechanochemical cycle. Dynein is anchored to the A‐tubule (not shown) via the N‐terminal domain and IC/LC complex. Nucleotide binding at AAA1 converts the motor to the pre‐power stroke I conformation in which the linker (purple) undergoes an almost 90° bend causing the AAA ring to swing, simultaneously the microtubule‐binding domain (MTBD) adopts a low affinity conformation and releases the B‐tubule. Following nucleotide hydrolysis, the MTBD transitions to a high affinity state and rebinds the microtubule at a new site. Phosphate release triggers the return of the linker to its original unbent state and thus drives the attached microtubule toward the axonemal distal tip. Finally, ADP departs the active site leaving the AAA1 nucleotide pocket empty and the HC capable of undergoing another mechanochemical cycle. HC, heavy chain; IC, intermediate chain; LC, light chain [Color figure can be viewed at http://wileyonlinelibrary.com]

Dyneins are highly complex multimeric motors built around one to three ~540 kDa heavy chain (HC) motor units (King, 2018); understanding of the general properties of these massive proteins has come from studies on cytoplasmic and intraflagellar transport dyneins as well as those derived from the axoneme. Each HC consists of an N‐terminal region involved in assembly, interaction with other dynein components, and stable association with the outer doublet A‐tubule. This is followed by a linker region that crosses the plane of a six‐membered ring of AAA+ domains (AAA1–6) and which moves during a cycle of ATP binding, hydrolysis and product release at AAA1 to generate the power stroke (Burgess et al., 2003; Kon et al., 2005; Roberts et al., 2009; Carter et al., 2011; Kon et al., 2012; Schmidt et al., 2014). The ATP‐dependent microtubule binding domain that interacts transiently with the outer doublet B‐tubule is located at the tip of an antiparallel coiled coil which emanates from the AAA4 domain and is supported by a second coiled coil “buttress” derived from AAA5. ATP‐driven changes in the coiled coil register control microtubule binding affinity (Carter et al., 2008; Redwine et al., 2012) and thus allow for coordination of the ATPase cycle with microtubule binding and release (Figure 1).

**Figure 2:**
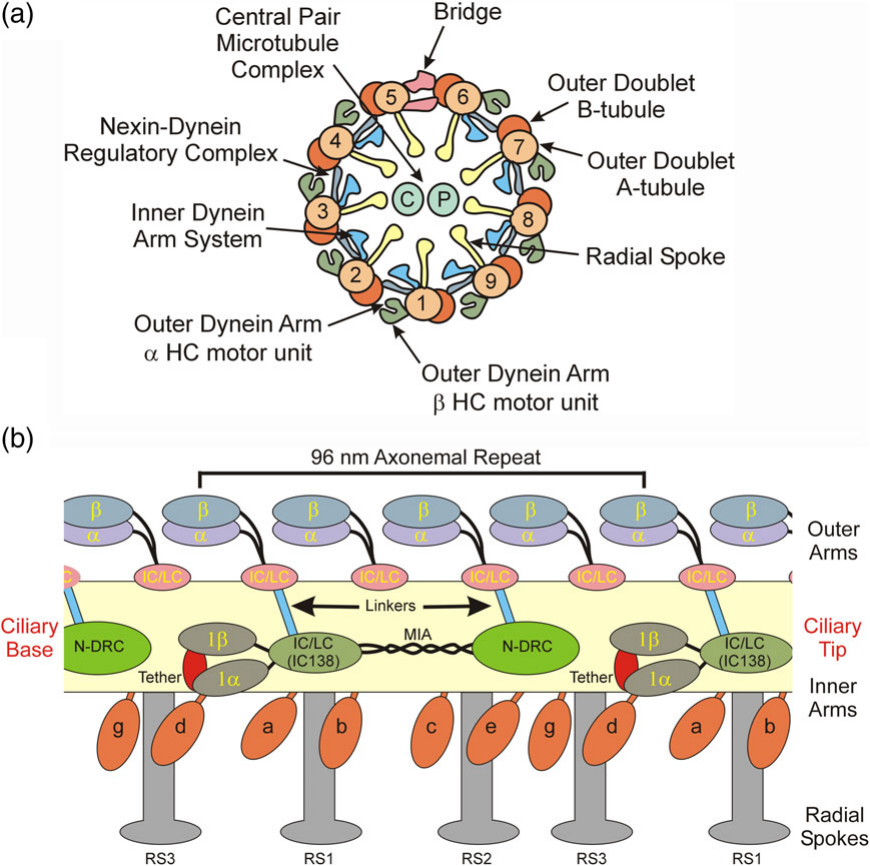
Arrangement and interconnectivity of axonemal motors and regulators. (a) Diagram of the ciliary axonemal cross‐section indicating the key structural elements including the A‐ and B‐tubules of the outer doublet microtubules, the inner and outer dynein arms, nexin‐dynein regulatory complex, radial spokes, central pair microtubule complex, and the bridge structure that spans the gap between doublets 5 and 6. Based on analysis of orthologous components in *Chlamydomonas*, within the sea urchin outer dynein arm the HCs are arranged with the β HC outermost. (b) diagram illustrating the general arrangement along the axonemal long axis of the inner and outer arm dynein motors and the key regulatory components that control their activity in sea urchin sperm; unlike opisthokonts, in *Chlamydomonas* the outer arms have three HCs and RS3 is truncated lacking the spoke head and most of the stalk. All these components are tightly associated with A‐tubule (yellow) and the orientation with respect to the ciliary base and tip is indicated. Inner arm I1/f consists of the 1α and 1β HC motors (indicated as 1α and 1β) and the IC/LC complex that contains the key regulator IC138. The motor domains of the I1/f inner arm dynein are attached to the A‐tubule via the tether/tether head complex (red) that restricts their motion. Dyneins transiently interact with the B‐tubule (not shown) in an ATP‐dependent manner to generate force, while the N‐DRC provides a nucleotide‐independent linkage that connects adjacent doublets and acts to resist sliding. This converts the dynein‐generated unidirectional force vector into a microtubule bend. The radial spokes transduce mechanical signals from the central pair microtubule complex (not shown) to the inner arm system. Not shown is the calmodulin spoke complex that interconnects RS2, RS3, and the N‐DRC. Linkers (blue) allow signal transmission from inner arm I1/f and the N‐DRC (green) to the outer arm dyneins. HC, heavy chain; IC, intermediate chain; LC, light chain; N‐DRC, nexin‐dynein regulatory complex [Color figure can be viewed at http://wileyonlinelibrary.com]

Within the axoneme three distinct dynein “types” are evident built into the 96‐nm axonemal repeat unit (Figure 2a,b). The outer arms contain two or three different HCs depending on species (opisthokonts have two, while chlorophyte algae and alveolates have three; Wickstead, 2018) with a variety of associated intermediate and light chains (ICs and LCs, respectively), and occur every 24 nm along the axoneme (King, 2018). These motors provide much of the power output for the organelle, and loss of these dyneins results in a characteristic reduction in beat frequency but does not alter waveform (Kamiya & Okamoto, 1985; Mitchell & Rosenbaum, 1985). Within the inner arm system, each 96‐nm repeat begins with a two‐HC inner arm I1/f that plays a key role in waveform determination and which is the subject of several signaling inputs that control activity (Porter & Sale, 2000). This is followed by six different monomeric HC dyneins arranged in pairs (dyneins a/b, c/e, and g/d) around the base of the three radial spokes (RS1–3) which protrude into the axonemal lumen toward the central pair microtubule complex (note that in *Chlamydomonas*, RS3 is present in a truncated form lacking the spoke head and most of the shaft). Each monomeric dynein within the repeat is distinct and indeed they have different motor and ATPase properties (Kagami & Kamiya, 1992). Although all monomeric dyneins contain a single molecule of actin, one dynein in each radial spoke cluster (dyneins a, c, and d) contains a light chain dimer (termed p33 in sea urchin or p28 in *Chlamydomonas*), while the other (dyneins b, e, and g) has the Ca^2+^‐binding calmodulin homologue centrin (King, 2018); dynein d is also unusual in that it contains two additional components—p44 containing tetratricopeptide repeats and p38 which may mediate docking at a specific axonemal location (Yamamoto et al., 2008).

Not only does the axonemal repeat unit contain multiple different dyneins, but also several structurally complex multi‐component systems that are tightly bound to the axoneme and which allow for the rapid propagation of regulatory signals (Figure 2a,b). The tether/tether head complex interacts with the AAA rings of both I1/f dynein HCs, one monomeric dynein (dynein d) from the previous repeat unit and the A‐tubule (Fu et al., 2018; Kubo et al., 2018). This complex restricts the motion of the I1/f dynein AAA rings, may sense mechanical strain and is thought to provide a signal transduction pathway from RS3 through the I1/f dynein and potentially impacting a second monomeric dynein (dynein a) that associates with the base of RS1. The nexin‐dynein regulatory complex (N‐DRC) is a key multimeric axonemal regulator located on the A‐tubule near the base of RS2 and RS3 and monomeric dyneins c/e and g/d, and provides an ATP‐independent linkage to the B‐tubule (Gardner et al., 1994; Heuser et al., 2009; Awata et al., 2015; Porter, 2018). The N‐DRC is connected to the IC/LC complex of dynein I1/f by the “modifier of inner arms” (MIA) complex which consists of two coiled coil proteins (Yamamoto et al., 2013). A further regulatory unit—the calmodulin spoke complex (CSC)—connects RS2, RS3, and the N‐DRC (Dymek et al., 2011; Heuser et al., 2012). Furthermore, efficient ciliary beating of course requires the coordinated activity of both the inner and outer dynein arm systems. It is now clear that two linkages directly connect these motor arrays: one linker attaches the IC/LC complex of inner arm I1/f to the first of the four outer arms in the axonemal repeat, while a second linker bridges the gap between the N‐DRC and the third outer arm within the same repeat (Nicastro et al., 2006; Oda et al., 2013). Thus, there are direct structural connections linking the key axonemal motors and regulators which allow for both mechanical and biochemical inputs and rapid directed signal propagation throughout this intricately interconnected system.

Importantly, in sea urchin sperm axonemes a bridging structure (termed o‐SUB5–6), rather than dynein arms, connects doublet microtubules 5 and 6 (Lin et al., 2012). The bridge is thought to form a rigid component within the nine outer doublets that runs essentially parallel to the plane of the central pair microtubule complex whose orientation in sea urchin sperm is also enforced by inelastic linkers. Combined, these two structural specializations are predicted to only allow flagellar bending in a plane that is perpendicular to the o‐SUB5–6 bridge presumably by providing an inherent structural impediment to out‐of‐plane movement. In *Chlamydomonas*, a bridge structure is located between doublets 1 and 2 (Hoops & Witman, 1983). However, the central pair complex is not held rigid but rather rotates driven by the propagating bend (Omoto et al., 1999; Mitchell & Nakatsugawa, 2004); potentially this allows cilia to generate waveforms with varying effective stroke directions, rather than being entrained in a single plane.

## 3 PRE‐/POST‐POWERSTROKE CONFORMATIONAL STATES AND RELATIONSHIP TO THE DYNEIN MECHANOCHEMICAL CYCLE

Dyneins undergo several structural transitions during a mechanochemical cycle (Burgess et al., 2003; Roberts et al., 2009; Schmidt et al., 2012; Cianfrocco et al., 2015; Schmidt and Carter, 2018) that can be differentiated by cryo‐ET (Lin & Nicastro, 2018). These include: (a) changes in the linker position and its internal conformation with respect to the AAA domain head; (b) alterations in orientation of the microtubule‐binding stalk with respect to the axonemal long axis which correlates with movement away from the B‐tubule; and (c) changes in affinity of the microtubule‐binding domain for the outer doublet B‐tubule that are reflected by tightly bound or microtubule‐released states.

Each of these transitions is connected to the ATPase cycle (Figure 1). In the post‐power stroke conformation, a dynein HC has an empty ATP binding site at AAA1, a linker that is in the straight conformation and traverses the plane of the AAA ring from AAA1, from which it emanates, to AAA4, and a microtubule‐binding domain in the high affinity state tightly bound to the B‐tubule. Binding of ATP at AAA1 drives a transition to the pre‐power stroke I conformation in which the linker becomes bent by almost 90° as it passes AAA1 and now exits the AAA ring at AAA2. Simultaneously, the microtubule‐binding domain changes to the low affinity conformation, releases the B‐tubule and the entire microtubule‐binding stalk moves toward the ciliary base and thus away from the B‐tubule. A second pre‐power stroke conformation is also seen in the tomograms and presumably derives from hydrolysis of ATP to ADP + P_i_. Here the linker remains bent, but the microtubule‐binding domain reconnects with the B‐tubule where it can now rebind in a high affinity state. The actual power stroke that generates microtubule sliding toward the axonemal distal tip is triggered by phosphate release that promotes a conformational change in the linker back to its unbent state, leading to rotation of the AAA ring to its original orientation and thereby winching the microtubule toward the ciliary tip. Finally, ADP release from AAA1 then allows for ATP rebinding and a new mechanochemical cycle and power stroke.

## 4 DYNEIN CONFORMATION IN IMMOTILE AXONEMES AND INHIBITED SPERM

What is the power stroke status of dynein motors in cilia that are not moving? In the sea urchin sperm system used by Lin and Nicastro (2018), immotile structures can be obtained in one of two ways. Either they can simply be demembranated in a buffer that lacks ATP; in this case, all dyneins should lack nucleotide at AAA1 and thus be in the post‐power stroke conformation. Alternatively, intact live sperm can be treated with a membrane‐permeable adenine analog, erytho‐9‐(2‐hydroxynonyl)adenine (EHNA), which completely inhibits motility (Bouchard et al., 1981). EHNA is not a simple competitive inhibitor of dynein ATPase activity, rather it acts as a mixed (non‐competitive) inhibitor affecting both *V*_max_ and *K*_m_ suggesting that it may interact with a site distinct from the ATP‐binding/hydrolytic site on AAA1. Based on a structural analogy with EHNA‐bound adenosine deaminase, Steinman and Kapoor (2018) proposed that EHNA enters the AAA1 ATP‐binding site, but through its long alcohol chain also associates with nearby residues outside the nucleotide pocket resulting in complex inhibition kinetics; this prediction remains untested. Another, not mutually exclusive, possibility is that it may replace nucleotide associated with AAA3 which appears important for mechanochemical coupling. Furthermore, it is quite possible that EHNA associates with other ciliary components that normally bind adenosine‐containing compounds, making understanding its mode of action in live cells complicated.

Outer arm dynein HCs in both EHNA‐treated sperm and demembranated axonemes were all found in the post‐power stroke (inactive) state tightly bound to the B‐tubule. Not only was this the case in straight regions, but also in segments of the axonemes or inhibited sperm tails that were passively bent, presumably by fluid forces imparted during sample preparation. Similar results were obtained for the inner arm dyneins in both EHNA‐inhibited sperm and ATP‐depleted axonemes, indicating that in situ all motors undergo nucleotide release and can achieve the post‐power stroke conformation in both straight and curved axonemal regions.

This result raises the intriguing question of how dynein power stroke status correlates with axonemal stiffness, as EHNA‐treated sand dollar sperm have a significantly reduced flexural rigidity (0.19 × 10^−21^ Nm^2^) compared to either demembranated (14 × 10^−21^ Nm^2^) or live, actively beating, sperm (Ishijima & Hiramoto, 1994). Potentially, the observed post‐power stroke states induced by demembranation and EHNA treatment may be functionally different in a manner not readily discernable by cryo‐ET.

## 5 DYNEIN POWER STROKE STATES VARY DURING ACTIVE BEATING

A major surprise from the Lin and Nicastro (2018) study is that on examination of actively beating sperm, most outer arm dyneins (~85%) were found in a pre‐power stroke conformation with a primed linker. The sea urchin outer arm dynein contains two HC motors termed α and β (these are equivalent to the γ and β HCs of *Chlamydomonas*, respectively; Wickstead & Gull, 2007; Hom et al., 2011). Intriguingly, the α HC was in the microtubule‐detached pre‐power stroke I conformation, while the β HC was in pre‐power stroke II and tightly bound to the adjacent B‐tubule. Thus, although the activity of these two motors is apparently coordinated, they may function out of phase with each other; attachment of only a single outer arm dynein motor unit to the outer doublet B‐tubule was also observed previously by Ueno et al. (2008). Not only do these two HCs have different inherent motor properties (Sale & Fox, 1988; Moss et al., 1992; Moss et al., 1992), the α HC but not the β HC is thought to associate with a highly conserved leucine‐rich repeat light chain (termed LC2 in sea urchin, DNAL1 in mammals and LC1 in *Chlamydomonas*) that, at least in the equivalent *Chlamydomonas* γ HC, is located at the tip of the microtubule‐binding stalk, interacts with tubulin and might alter or modify mechanochemical coupling (Wu et al., 2000; Patel‐King & King, 2009; King & Patel‐King, 2012; Ichikawa et al., 2015).

In addition to the pre‐power stroke conformations, three intermediate states (IM1–3) of the outer arm were also detected. In IM1, both HCs are in a post‐power stroke‐like state, whereas in IM2 and IM3 either the α (IM3) or β (IM2) HC is in a transitional conformation, while the other HC remains in the post‐power stroke‐like state. The microtubule binding stalks of these intermediate structures were not clearly defined in the tomograms due to positional variation from one dynein to the next and information loss during imaging averaging. However, they are thought to represent either microtubule‐detached or weakly bound states; clearly explicitly testing this prediction will be a key next step.

**Figure 3:**
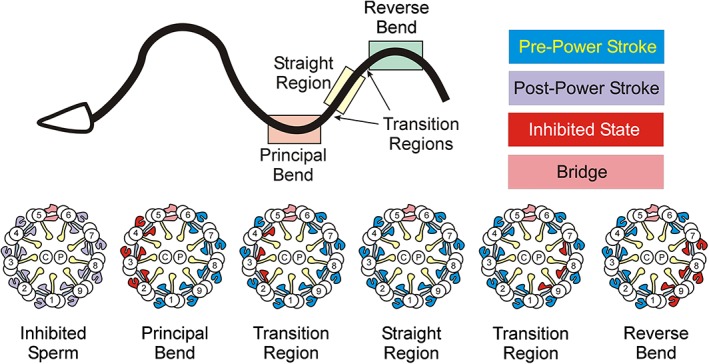
Dynein power stroke transitions during oscillatory beating. *Top* – Diagram of a swimming sea urchin sperm. The symmetric flagella waveform propagates from base to tip. Regions corresponding to the principal bend (light pink), reverse bend (light green), and the straight region (light yellow) between them are boxed. Also shown (arrows) are the “principal bend to straight region” and “straight region to reverse bend” transition segments where only some inner arms are in the inactive conformation. *Bottom* – Diagrams of axonemal cross‐sections showing the status of individual dynein motors on each doublet in EHNA‐inhibited sperm, principal and reverse bends, straight intervening region and the transitional segments; blue – pre‐power stroke, purple – post‐power stroke, red – inhibited (weakly bound or microtubule‐detached) state, pink – bridge structure between doublets 5 and 6, blue‐gray – N‐DRC, yellow – radial spokes. In bends, both inner and outer arms on doublets 2–4 or 7–9 are in the inhibited state. In the straight region, most dyneins are in a pre‐power stroke conformation, while in transitions between bends and straight segments, only inner arm subsets are inactivated. EHNA, erytho‐9‐(2‐hydroxynonyl)adenine; N‐DRC, nexin‐dynein regulatory complex. [Color figure can be viewed at http://wileyonlinelibrary.com]

The intermediate states of outer arm dyneins were almost exclusively found in curved regions associated with doublets 2–4 in principal bends and doublets 7–9 in reverse bends; these regions represent the inside of the axonemal curve, while essentially all dyneins on the opposing side of the curve were in the pre‐power stroke state (Figure 3). In contrast, in straight regions outer arm dyneins on all doublets were in the active pre‐power stroke conformation; the inner arms were somewhat variable and showed small clusters of dyneins in intermediate conformations surrounded by those in the pre‐power stroke state. This raises the important issue of whether dyneins in straight axonemal regions are all locked in the pre‐power stroke state by the enhanced load derived from dyneins on the opposite side of the axoneme adopting the same strain‐induced conformation, or if they are actively cycling although perhaps spending most time in the pre‐power stroke state; this would be consistent with kinetic measurements showing the power stroke/ADP release step(s) are rate‐limiting (Chilcote & Johnson, 1989). If locked in a tight microtubule binding conformation, for outer arms this must presumably be mediated by the β HC, as the α HC is in a low affinity pre‐power stroke I state. This is consistent with the observation that a *Chlamydomonas* mutant (*oda4‐s7*) lacking only the motor domain of the β HC has a motility phenotype similar to that observed in strains completely lacking the outer arm (Sakakibara et al., 1993), while a similar mutation in the γ HC *oda2‐t* mutant is less disruptive (Liu et al., 2008). Confusingly however, at least in vitro, the β HC exhibits a low duty ratio spending most of the mechanochemical cycle time in a detached state (Sakakibara & Nakayama, 1998).

Lin and Nicastro (2018) propose that axonemal dynein affinity for microtubules increases under load and may even generate a catch bond. Cytoplasmic dynein indeed responds to increased load by binding the microtubule more tightly (McKenney et al., 2010; Reddy et al., 2016), although for this motor the response requires an additional factor, Lis1. In *Chlamydomonas*, Lis1 levels are low and substoichiometric in cilia under normal swimming conditions; however, they increase significantly when cells are placed under high viscous load, and trafficked Lis1 binds the outer arm resulting in increased power output (Rompolas et al., 2012). Although the presence of Lis1 in sea urchin sperm flagella has not been described, murine tracheal and oviductal cilia which function in a high‐load mucus‐rich regime both contain this dynein regulatory factor (Pedersen et al., 2007). Alternatively, dynein‐microtubule interactions may be controlled by changing the nucleotide present in AAA3; when ADP is bound at this site, the cytoplasmic dynein HC undergoes a standard mechanochemical cycle, whereas with ATP in AAA3, coupling between AAA1 and the microtubule‐binding domain is disrupted and microtubule release diminished (Kon et al., 2004; Dewitt et al., 2015; Nicholas et al., 2015; Schmidt & Carter, 2018). Given the essentially all‐or‐none behavior of outer arms and the more variable states of the inner arm system observed by Lin and Nicastro (2018), straight axonemal regions may contain a complex admixture of mainly stalled dyneins comingled with small subsets of actively cycling inner arms.

Together, these observations provide direct support for two key tenets of the switch‐point hypothesis—namely, that dyneins on opposite sides of a curved region are in different functional states, and that this functional dichotomy switches with bend direction. Furthermore, they support an interpretation of dynein activity whereby axonemal motors are inherently in the active conformation, and that bend initiation and propagation is a result of a subset transitioning to a microtubule‐detached or inhibited state which disrupts the force balance allowing for active sliding driven by dyneins on the uninhibited side of the axoneme. This is consistent with the observations of Shingyoji et al. (1977) who demonstrated that dynein‐driven sliding causes bending, and Brokaw (1991) who found that sliding occurs in bends. Further support for this idea comes from the observation that in paralyzed rigid flagella from a *Chlamydomonas* mutant lacking the central pair microtubule complex, outer arms are all in the pre‐power stroke state (Lin & Nicastro, 2018); that is*,* they are forced‐balanced and immotile presumably because they cannot initiate the inhibitory signal that would allow bending.

Analysis of the inner arm system revealed a more complex response. In both inhibited sperm and ATP‐depleted axonemes, these dyneins were in states similar to outer arms. Furthermore, in actively beating sperm they mostly adopted a pre‐power stroke state, although with a higher degree of variability than observed for outer arms. However, two distinct responses were observed in curved regions. The two‐HC I1/f dynein and three of the monomeric HC dyneins (dyneins a, g, and d) were found in intermediate or inhibited states on doublets that correspond to those observed for outer arms. This suggests that both outer arms and these specific inner arms, the I1/f dynein and monomeric dyneins associated with RS1 (dynein a) and RS3 (dyneins g and d), may respond to the same, currently unknown, inhibitory signal. However, coupling between this inhibitory signal and the inner arm system is more nuanced as some small groups of inner arms on only one or two doublets in “transition” regions immediately adjacent to curves were also in the microtubule‐detached inhibited state. As rapidly frozen sperm flagella in essence provide a temporal snapshot of dynein activity during wave propagation, the presumption is that these are motors in the process of initiating the ciliary bend or recovering from it. One possible confounding issue here is that during preparation of the actively swimming sperm samples, most liquid must be removed from the electron microscopy grid immediately prior to plunge freezing, which might impart high hydrodynamic forces for a sufficient period that it could alter dynein activity. Using a mutant lacking the motor domain of the I1/f dynein β HC, Lin and Nicastro (2018) also observed hyperphosphorylation of the key phospho‐regulatory subunit IC138 (Porter & Sale, 2000; Hendrickson et al., 2004), thereby providing a connection between enzymatic signal transduction through the I1/f dynein and axonemal conformation.

Intriguingly, three other monomeric HC dyneins that are adjacent within the 96‐nm axonemal repeat—one that associates with RS1 (dynein b) and both surrounding RS2 (dyneins c and e)—did not exhibit inhibited states that corresponded to bend direction. These dyneins therefore may not be essential for initiating or propagating a waveform but rather provide either regulatory inputs or allow responses to altered hydrodynamic or other external conditions. For example, dynein c is a very fast microtubule motor in vitro (~16 μm/s) and dynein c‐mediated microtubule translocation velocity is significantly enhanced by the presence of the inherently slow (~1 μm/s) dynein e motor (Shimizu et al., 2014). *Chlamydomonas* mutants lacking dynein c have approximately normal swimming behavior under standard solution conditions but a greatly reduced ability to generate propulsive force when the medium viscosity is increased (Yagi et al., 2005). Thus, the powerful dynein c, presumably aided by the adjacent dynein e, provides a built‐in response mechanism within the inner arm system, perhaps signal coordinated through RS2, for modulating power production as environmental fluid properties change. It would be interesting to test if the conformation of these monomeric dyneins correlate with bend formation when sperm are under higher viscous load.

## 6 CONTROLING DYNEIN STATE TRANSITIONS TO GENERATE BENDS

The structural data of Lin and Nicastro (2018) reveal changes in the organization of axonemal regulatory substructures and predict a pathway for regulatory signal propagation. These involve the stalks of the I1/f dynein HCs connecting to the nexin linker from the N‐DRC (1α HC) or dynein g (1β HC) located next to RS3 (from the previous axonemal repeat), and the I1/f HC tether complex again connecting to RS3 and to the I1/f dynein IC/LC complex which contains the key phospho‐regulator IC138. The “switch inhibition” model requires that an inhibitory signal leading to dynein release from the B‐tubule propagate along the axoneme and change doublets to allow for both principal and reverse bends; this inhibitory signal must also be turned off appropriately. Furthermore, an essential component of the switch inhibition model is that it can provide an “all or none” response, where force imbalance leading to microtubule sliding only occurs once a sufficient number of dynein motors have been inhibited.

Previously, four general models have been proposed to account for asymmetric dynein activity in beating cilia:

1. The “distributor” model in which signals propagate from the central pair microtubule complex through the radial spokes and N‐DRC to affect inner arms and subsequently outer arms. This transduction pathway is thought to include both direct mechanical interactions (e.g.*,* between the radial spoke head and the central pair; Oda et al., 2014) as well as chemical modifications (Smith & Yang, 2004).
2. The “sliding control” model where increasing load leads to more rapid dynein release from the B‐tubule, so that dynein force output goes down as the sliding rate increases (Brokaw, 1971).
3. The “curvature control” model was originally proposed by Brokaw (1975). In the most recent formulation of curvature control, dyneins are proposed to detect alterations in the rate of change in axonemal curvature (Sartori et al., 2016).
4. The “geometric clutch” model which predicts that changes in interdoublet microtubule distances control whether or not dyneins are actively engaged with the neighboring B‐tubule (Lindemann, 2002).

There is considerable general support for the “distributor” model based on genetic, biochemical and structural data, and the Lin and Nicastro (2018) results are certainly consistent with this hypothesis. However, Lin and Nicastro (2018) also observed small changes in interdoublet microtubule spacing in those parts of bent axonemes where the outer arms were in intermediate conformations. Potentially this spacing increase might induce dynein‐microtubule release and so the current data also fit predictions for the geometric clutch, keeping in mind that the spacing change may be a consequence of dynein release rather than causative. Although the current results do not appear compatible with sliding control, curvature control where dyneins on one side of the axoneme release from the microtubule at a set rate of change in bend angle might also be consistent with the structural data. Direct tests for ensemble axonemal dynein motor function during variations in defined load and/or perceived curvature combined with computational approaches will be needed to further address this issue. Thus, it appears reasonable to conclude that a complete understanding of axonemal dynein control may require incorporating elements from several of these different models.

The results of the Lin and Nicastro (2018) study now pose a major question ‐ what is the nature of the inhibitory signal and how is its propagation controlled? The model suggests that these signals first result in inner arm dyneins transitioning to an inhibited state on either doublets 2–4 or 7–9, and that on passing a critical threshold number, opposing dyneins in the pre‐power stroke state can generate enough force to initiate microtubule sliding and bend formation. Transition from bend initiation to a complete bend might be driven either by transmission of the inhibitory signal to the outer arms which provide most of the force output and/or by these dyneins responding directly to the increasing curvature; that is, different dynein subsets might respond to distinct inhibitory signals. To allow for recovery and axonemal straightening, the inhibitory signal(s) must be turned off before a bend in the opposite direction can be initiated.

Although most motile cilia contain inner arms which could be inhibited to initiate bending, there are examples of these motile organelles that only have outer arms; for example, the flagellated motile male gametes of the centric diatom *Thalassiosira* which lack inner arms, radial spokes and the central pair complex (Armbrust et al., 2004). Understanding how individual outer arm HCs in this organism respond during transitions to initiate or recover from bending might provide insight into their out‐of‐phase behavior and the general mechanisms of their control.

Structural studies of canonical cytoplasmic dynein and the dynein that powers retrograde intraflagellar transport revealed an auto‐inhibited state (the φ particle), in which two motor heads stack together forming an interface that buries the linkers that cross the AAA ring and in which the microtubule‐binding stalks are crossed, thus structurally inhibiting the main mechanical elements (Torisawa et al., 2014; Pigino & King, 2017; Toropova et al., 2017; Zhang et al., 2017). This structure is not observed in axonemal dyneins in situ, and thus the inhibited axonemal dynein state seen by Lin and Nicastro (2018) has a distinct structural basis. Whether the φ particle conformation might occur in cytoplasm where axonemal dyneins are stored, or after transport into the cilium but prior to axonemal incorporation remains an open question. For example, axonemal dyneins stockpiled in cytoplasm would presumably move to the minus end of any microtubule they encountered unless inhibited in some manner. Similarly, motor activity by recently trafficked undocked axonemal dyneins within cilia would be energetically unfavorable and likely result in motor accumulations at the ciliary base.

Finally, although oscillatory bending with either planar or three‐dimensional waveforms are the most common forms of ciliary motion, other modalities occur. For example, cilia at the embryonic node beat with a vortical motion sweeping out a cone‐shaped volume through rotational movement apparently driven by motors near the axonemal base (Nonaka et al., 2005; Okada et al., 2005). Potentially a modified switch inhibition model where the inhibitory signal transits circumferentially from doublet to doublet in a restricted region near the ciliary base, rather than moving longitudinally toward the ciliary tip, might result in the observed motion.

In conclusion, the cryo‐ET studies of Lin and Nicastro (2018) have provided an unprecedented view of dynein conformational states during active ciliary beating and lead to a dramatic change in our understanding of how waveforms are generated. In the future, it will be important to clearly define the structural conformation of the intermediate states in which dyneins are interpreted as inhibited or microtubule‐released. Similarly, determining how these altered states correlate with changes in shear angle along a beating flagellum will provide additional mechanistic insight into oscillatory motion. Clearly, some of the next key issues to solve include understanding the nature of the signal that disrupts axonemal dynein force balance and the manner in which it is propagated and switched between different sets of microtubule doublets.

## References

[cm21483-bib-0001] Armbrust, E. V. , Berges, J. A. , Bowler, C. , Green, B. R. , Martinez, D. , Putnam, N. H. , … Rokhsar, D. S. (2004). The genome of the diatom *Thalassiosira pseudonana*: Ecology, evolution, and metabolism. Science, 306, 79–86. 15459382 10.1126/science.1101156

[cm21483-bib-0002] Awata, J. , Song, K. , Lin, J. , King, S. M. , Sanderson, M. J. , Nicastro, D. , & Witman, G. B. (2015). DRC3 connects the N‐DRC to dynein g to regulate flagellar waveform. Molecular Biology of the Cell, 26, 2788–2800. 26063732 10.1091/mbc.E15-01-0018 PMC4571338

[cm21483-bib-0003] Bayly, P. V. , & Dutcher, S. K. (2016). Steady dynein forces induce flutter instability and propagating waves in mathematical models of flagella. Journal of the Royal Society Interface, 13, 0523. 10.1098/rsif.2016.0523 PMC5095214 27798276

[cm21483-bib-0004] Bouchard, P. , Penningroth, S. M. , Cheung, A. , Gagnon, C. , & Bardin, C. W. (1981). Erythro‐9‐[3‐(2‐hydroxynonyl)]adenine is an inhibitor of sperm motility that blocks dynein ATPase and protein carboxylmethylase activities. Proceedings of the National Academy of Sciences of the United States of America, 78, 1033–1036. 6453342 10.1073/pnas.78.2.1033 PMC319940

[cm21483-bib-0005] Brokaw, C. (1991). Microtubule sliding in swimming sperm flagella: Direct and indirect measurements on sea urchin and tunicate spermatozoa. Journal of Cell Biology, 114, 1201–1215. 1894694 10.1083/jcb.114.6.1201 PMC2289132

[cm21483-bib-0006] Brokaw, C. (2009). Thinking about flagellar oscillation. Cell Motility and the Cytoskeleton, 66, 425–436. 18828155 10.1002/cm.20313

[cm21483-bib-0007] Brokaw, C. J. (1971). Bend propagation by a sliding filament model for flagella. Journal of Experimental Biology, 55, 289–304. 5114025 10.1242/jeb.55.2.289

[cm21483-bib-0008] Brokaw, C. J. (1975). Molecular mechanism for oscillation in flagella and muscle. Proceedings of the National Academy of Sciences of the United States of America, 72, 3102–3106. 1059095 10.1073/pnas.72.8.3102 PMC432928

[cm21483-bib-0009] Burgess, S. A. , Walker, M. L. , Sakakibara, H. , Knight, P. J. , & Oiwa, K. (2003). Dynein structure and power stroke. Nature, 421, 715–718. 12610617 10.1038/nature01377

[cm21483-bib-0010] Carter, A. P. , Cho, C. , Jin, L. , & Vale, R. (2011). Crystal structure of the dynein motor domain. Science, 331, 1159–1165. 21330489 10.1126/science.1202393 PMC3169322

[cm21483-bib-0011] Carter, A. P. , Garbarino, J. E. , Wilson‐Kubalek, E. M. , Shipley, W. E. , Cho, C. , Milligan, R. A. , … Gibbons, I. R. (2008). Structure and functional role of dynein's microtubule‐binding domain. Science, 322, 1691–1695. 19074350 10.1126/science.1164424 PMC2663340

[cm21483-bib-0012] Chilcote, T. J. , & Johnson, K. A. (1989). Microtubule‐dynein cross‐bridge cycle and the kinetics of 5′‐adenylyl imidophosphate (AMP‐PNP) binding In Warner F. D., Satir P., & Gibbons I. R. (Eds.), Cell movement. The dynein ATPases (Vol. 1, pp. 235–243). New York, NY: Alan R. Liss.

[cm21483-bib-0013] Cianfrocco, M. A. , DeSantis, M. E. , Leschziner, A. E. , & Reck‐Peterson, S. L. (2015). Mechanism and regulation of cytoplasmic dynein. Annual Review of Cell and Developmental Biology, 31, 83–108. 10.1146/annurev-cellbio-100814-125438 PMC4644480 26436706

[cm21483-bib-0014] Dewitt, M. A. , Cypranowska, C. A. , Cleary, F. B. , Belyy, V. , & Yildiz, A. (2015). The AAA3 domain of cytoplasmic dynein acts as a switch to facilitate microtubule release. Nature Structural and Molecular Biology, 22, 73–80. 10.1038/nsmb.2930 PMC4286497 25486306

[cm21483-bib-0015] Dymek, E. E. , Heuser, T. , Nicastro, D. , & Smith, E. F. (2011). The CSC is required for complete radial spoke assembly and wild‐type ciliary motility. Molecular Biology of the Cell, 22, 2520–2531. 21613541 10.1091/mbc.E11-03-0271 PMC3135477

[cm21483-bib-0016] Fu, G. , Wang, Q. , Phan, N. , Urbanska, P. , Joachimiak, E. , Lin, J. , … Marshall, W. (2018). The I1 dynein‐associated tether and tether head complex is a conserved regulator of ciliary motility. Molecular Biology of the Cell, 29, 1048–1059. 29514928 10.1091/mbc.E18-02-0142 PMC5921572

[cm21483-bib-0017] Gardner, L. C. , O'Toole, E. , Perrone, C. A. , Giddings, T. , & Porter, M. E. (1994). Components of a "dynein regulatory complex" are located at the junction between the radial spokes and the dynein arms in *Chlamydomonas* flagella. Journal of Cell Biology, 127, 1311–1325. 7962092 10.1083/jcb.127.5.1311 PMC2120243

[cm21483-bib-0018] Hendrickson, T. W. , Perrone, C. A. , Griffin, P. , Wuichet, K. , Mueller, J. , Yang, P. , … Sale, W. S. (2004). IC138 is a WD‐repeat dynein intermediate chain required for light chain assembly and regulation of flagellar bending. Molecular Biology of the Cell, 15, 5431–5442. 15469982 10.1091/mbc.E04-08-0694 PMC532023

[cm21483-bib-0019] Heuser, T. , Dymek, E. E. , Lin, J. , Smith, E. F. , & Nicastro, D. (2012). The CSC connects three major axonemal complexes involved in dynein regulation. Molecular Biology of the Cell, 23, 3143–3155. 22740634 10.1091/mbc.E12-05-0357 PMC3418309

[cm21483-bib-0020] Heuser, T. , Raytchev, M. , Krell, J. , Porter, M. E. , & Nicastro, D. (2009). The dynein regulatory complex is the nexin link and a major regulatory node in cilia and flagella. Journal of Cell Biology, 187, 921–933. 20008568 10.1083/jcb.200908067 PMC2806320

[cm21483-bib-0021] Hom, E. , Witman, G. B. , Harris, E. H. , Dutcher, S. K. , Kamiya, R. , Mitchell, D. R. , … King, S. M. (2011). A unified taxonomy for ciliary dyneins. Cytoskeleton, 68, 555–565. 21953912 10.1002/cm.20533 PMC3222151

[cm21483-bib-0022] Hoops, H. J. , & Witman, G. B. (1983). Outer doublet heterogeneity reveals structural polarity related to beat direction in *Chlamydomonas* flagella. Journal of Cell Biology, 97, 902–908. 6224802 10.1083/jcb.97.3.902 PMC2112583

[cm21483-bib-0023] Ichikawa, M. , Saito, K. , Yanagisawa, H.‐A. , Yagi, T. , Kamiya, R. , Yamaguchi, S. , … Toyoshima, Y. Y. (2015). Axonemal dynein light chain‐1 locates at the microtubule‐binding domain of the γ heavy chain. Molecular Biology of the Cell, 26, 4236–4247. 26399296 10.1091/mbc.E15-05-0289 PMC4642857

[cm21483-bib-0024] Ishijima, S. , & Hiramoto, Y. (1994). Flexural rigidity of echinoderm sperm flagella. Cell Structure and Function, 19, 349–362. 7720094 10.1247/csf.19.349

[cm21483-bib-0025] Kagami, O. , & Kamiya, R. (1992). Translocation and rotation of microtubules caused by multiple species of *Chlamydomonas* inner‐arm dynein. Journal of Cell Science, 103, 653–664.

[cm21483-bib-0026] Kamiya, R. , & Okamoto, M. (1985). A mutant of *Chlamydomonas reinhardtii* that lacks the flagellar outer dynein arm but can swim. Journal of Cell Science, 74, 181–191. 4030906 10.1242/jcs.74.1.181

[cm21483-bib-0027] King, S. M. (2018). Composition and assembly of axonemal dyneins In King S. M. (Ed.), Dyneins: Structure, biology and disease. Volume 1 ‐ The Biology of Dynein Motors (pp. 163–201). Oxford: Elsevier, Academic Press.

[cm21483-bib-0028] King, S. M. , & Patel‐King, R. S. (2012). Functional architecture of the outer arm dynein conformational switch. Journal of Biological Chemistry, 287, 3108–3122. 22157010 10.1074/jbc.M111.286211 PMC3270967

[cm21483-bib-0029] King, S. M. , & Sale, W. S. (2018). Fifty years of microtubule sliding in cilia. Molecular Biology of the Cell, 29, 698–701. 29535180 10.1091/mbc.E17-07-0483 PMC6003218

[cm21483-bib-0030] Kon, T. , Mogami, T. , Ohkura, R. , Nishiura, M. , & Sutoh, K. (2005). ATP hydrolysis cycle‐dependent tail motions in cytoplasmic dynein. Nature Structural and Molecular Biology, 12, 513–519. 10.1038/nsmb930 15880123

[cm21483-bib-0031] Kon, T. , Nishiura, M. , Ohkura, R. , Toyoshima, Y. Y. , & Sutoh, K. (2004). Distinct functions of nucleotide‐binding/hydrolysis sites in the four AAA modules of cytoplasmic dynein. Biochemistry, 43, 11266–11274. 15366936 10.1021/bi048985a

[cm21483-bib-0032] Kon, T. , Oyama, T. , Shimo‐Kon, R. , Imamula, K. , Shima, T. , Sutoh, K. , & Kurisu, G. (2012). The 2.8 Å crystal structure of the dynein motor domain. Nature, 484, 345–350. 22398446 10.1038/nature10955

[cm21483-bib-0033] Kubo, T. , Hou, Y. , Cochran, D. A. , Witman, G. B. , Oda, T. , & Zhu, X. (2018). A microtubule‐dynein tethering complex regulates the axonemal inner dynein f (I1). Molecular Biology of the Cell, 29, 1060–1074. 29540525 10.1091/mbc.E17-11-0689 PMC5921573

[cm21483-bib-0034] Lin, J. , Heuser, T. , Song, K. , Fu, X. , & Nicastro, D. (2012). One of the nine doublet microtubules of eukaryotic flagella exhibits unique and partially conserved structures. PLoS One, 7, e46494. 23071579 10.1371/journal.pone.0046494 PMC3468612

[cm21483-bib-0035] Lin, J. , & Nicastro, D. (2018). Asymmetric distribution and spatial switching of dynein activity generates ciliary motility. Science, 360, eaar1968. 29700238 10.1126/science.aar1968 PMC6640125

[cm21483-bib-0036] Lindemann, C. B. (2002). Geometric clutch model version 3: The role of the inner and outer arm dyneins in the ciliary beat. Cell Motility and the Cytoskeleton, 52, 242–254. 12112138 10.1002/cm.10049

[cm21483-bib-0037] Liu, Z. , Takazaki, H. , Nakazawa, Y. , Sakato, M. , Yagi, T. , Yasunaga, T. , … Kamiya, R. (2008). Partially functional outer arm dynein in a novel *Chlamydomonas* mutant expressing a truncated γ heavy chain. Eukaryotic Cell, 7, 1136–1145. 18487347 10.1128/EC.00102-08 PMC2446680

[cm21483-bib-0038] McKenney, R. J. , Vershinin, M. , Kunwar, A. , Vallee, R. B. , & Gross, S. P. (2010). Lis1 and NudE induce a persistent dynein force‐producing state. Cell, 141, 304–314. 20403325 10.1016/j.cell.2010.02.035 PMC2881166

[cm21483-bib-0039] Mitchell, D. R. , & Nakatsugawa, M. (2004). Bend propagation drives central pair rotation in *Chlamydomonas reinhardtii* flagella. Journal of Cell Biology, 166, 709–715. 15337779 10.1083/jcb.200406148 PMC1361683

[cm21483-bib-0040] Mitchell, D. R. , & Rosenbaum, J. L. (1985). A motile *Chlamydomonas* flagellar mutant that lacks outer dynein arms. Journal of Cell Biology, 100, 1228–1234. 3156867 10.1083/jcb.100.4.1228 PMC2113766

[cm21483-bib-0041] Moss, A. G. , Gatti, J. L. , & Witman, G. B. (1992). The motile beta/IC1 subunit of sea urchin sperm outer arm dynein does not form a rigor bond. Journal of Cell Biology, 118, 1177–1188. 1387405 10.1083/jcb.118.5.1177 PMC2289586

[cm21483-bib-0042] Moss, A. G. , Sale, W. S. , Fox, L. A. , & Witman, G. B. (1992). The alpha subunit of sea urchin sperm outer arm dynein mediates structural and rigor binding to microtubules. Journal of Cell Biology, 118, 1189–1200. 1387406 10.1083/jcb.118.5.1189 PMC2289587

[cm21483-bib-0043] Nicastro, D. , Schwartz, C. , Pierson, J. , Gaudette, R. , Porter, M. E. , & McIntosh, J. R. (2006). The molecular architecture of axonemes revealed by cryoelectron tomography. Science, 313, 944–948. 16917055 10.1126/science.1128618

[cm21483-bib-0044] Nicholas, M. P. , Berger, F. , Rao, L. , Brenner, S. , Cho, C. , & Gennerich, A. (2015). Cytoplasmic dynein regulates its attachment to microtubules *via* nucleotide state‐switched mechanosensing at multiple AAA domains. Proceedings of the National Academy of Sciences of the United States of America, 112, 6371–6376. 25941405 10.1073/pnas.1417422112 PMC4443381

[cm21483-bib-0045] Nonaka, S. , Yoshiba, S. , Watanabe, D. , Ikeuchi, S. , Goto, T. , Marshall, W. F. , & Hamada, H. (2005). *De novo* formation of left–right asymmetry by posterior tilt of nodal cilia. PLoS Biology, 3, e268. 16035921 10.1371/journal.pbio.0030268 PMC1180513

[cm21483-bib-0046] Oda, T. , Yagi, T. , Yanagisawa, H. , & Kikkawa, M. (2013). Identification of the outer‐inner dynein linker as a hub controller for axonemal dynein activities. Current Biology, 23, 656–664. 23583547 10.1016/j.cub.2013.03.028

[cm21483-bib-0047] Oda, T. , Yanagisawa, H. , Yagi, T. , & Kikkawa, M. (2014). Mechanosignaling between central apparatus and radial spokes controls axonemal dynein activity. Journal of Cell Biology, 204, 807–819. 24590175 10.1083/jcb.201312014 PMC3941055

[cm21483-bib-0048] Okada, Y. , Takeda, S. , Tanaka, Y. , Belmonte, J. ‐C. I. , & Hirokawa, N. (2005). Mechanism of nodal flow: A conserved symmetry breaking event in left‐right axis determination. Cell, 121, 633–644. 15907475 10.1016/j.cell.2005.04.008

[cm21483-bib-0049] Omoto, C. , Gibbons, I. , Kamiya, R. , Shingyoji, C. , Takahashi, K. , & Witman, G. (1999). Rotation of the central pair microtubules in eukaryotic flagella. Molecular Biology of the Cell, 10, 1–4. 9880321 10.1091/mbc.10.1.1 PMC25148

[cm21483-bib-0050] Patel‐King, R. S. , & King, S. M. (2009). An outer arm dynein light chain acts in a conformational switch for flagellar motility. Journal of Cell Biology, 186, 283–295. 19620633 10.1083/jcb.200905083 PMC2717645

[cm21483-bib-0051] Pedersen, L. , Rompolas, P. , Christensen, S. , Rosenbaum, J. L. , & King, S. M. (2007). The lissencephaly protein Lis1 is present in motile mammalian cilia and requires outer arm dynein for targeting to *Chlamydomonas* flagella. Journal of Cell Science, 120, 858–867. 17314247 10.1242/jcs.03374

[cm21483-bib-0052] Pigino, G. , & King, S. M. (2017). Switching dynein motors on and off. Nature Structural and Molecular Biology, 24, 557–559. 10.1038/nsmb.3429 28686228

[cm21483-bib-0053] Porter, M. E. (2018). Ciliary and flagellar motility and the nexin‐dynein regulatory complex In King S. M. (Ed.), Dyneins: Structure, biology and disease. Volume 1 ‐ The Biology of Dynein Motors (pp. 299–335). Oxford, UK: Elsevier.

[cm21483-bib-0054] Porter, M. E. , & Sale, W. S. (2000). The 9 + 2 axoneme anchors multiple inner arm dyneins and a network of kinases and phosphatases that control motility. Journal of Cell Biology, 151, F37–F42. 11086017 10.1083/jcb.151.5.f37 PMC2174360

[cm21483-bib-0055] Reddy, B. J. N. , Mattson, M. , Wynne, C. L. , Vadpey, O. , Durra, A. , Chapman, D. , … Gross, S. P. (2016). Load‐induced enhancement of dynein force production by Lis1–NudE *in vivo* and *in vitro* . Nature Communications, 7, 12259. 10.1038/ncomms12259 PMC4976208 27489054

[cm21483-bib-0056] Redwine, W. B. , Hernandez‐Lopez, R. , Zou, S. , Huang, J. , Reck‐Peterson, S. L. , & Leschziner, A. E. (2012). Structural basis for microtubule binding and release by dynein. Science, 337, 1532–1536. 22997337 10.1126/science.1224151 PMC3919166

[cm21483-bib-0057] Roberts, A. J. , Numata, N. , Walker, M. L. , Kato, Y. S. , Malkova, B. , Kon, T. , … Burgess, S. A. (2009). AAA+ ring and linker swing mechanism in the dynein motor. Cell, 136, 485–495. 19203583 10.1016/j.cell.2008.11.049 PMC2706395

[cm21483-bib-0058] Rompolas, P. , Patel‐King, R. S. , & King, S. M. (2012). Association of Lis1 with outer arm dynein is modulated in response to alterations in flagellar motility. Molecular Biology of the Cell, 23, 3554–3656. 22855525 10.1091/mbc.E12-04-0287 PMC3442404

[cm21483-bib-0059] Sakakibara, H. , & Nakayama, H. (1998). Translocation of microtubules caused by the αβ, β and γ outer arm dynein subparticles of *Chlamydomonas* . Journal of Cell Science, 111, 1155–1164. 9547292 10.1242/jcs.111.9.1155

[cm21483-bib-0060] Sakakibara, H. , Takada, S. , King, S. M. , Witman, G. B. , & Kamiya, R. (1993). A *Chlamydomonas* outer arm dynein mutant with a truncated β heavy chain. Journal of Cell Biology, 122, 653–661. 8335691 10.1083/jcb.122.3.653 PMC2119660

[cm21483-bib-0061] Sale, W. S. , & Fox, L. A. (1988). Isolated beta‐heavy chain subunit of dynein translocates microtubules *in vitro* . Journal of Cell Biology, 107, 1793–1797. 2972730 10.1083/jcb.107.5.1793 PMC2115335

[cm21483-bib-0062] Sartori, P. , Geyer, V. F. , Scholich, A. , Jülicher, F. , & Howard, J. (2016). Dynamic curvature regulation accounts for the symmetric and asymmetric beats of *Chlamydomonas* flagella. eLife, 5, e13258. 27166516 10.7554/eLife.13258 PMC4924999

[cm21483-bib-0063] Satir, P. (1968). Studies on cilia: III. Further studies on the cilium tip and a "sliding filament" model of ciliary motility. Journal of Cell Biology, 39, 77–94. 5678451 10.1083/jcb.39.1.77 PMC2107504

[cm21483-bib-0064] Schmidt, H. , & Carter, A. P. (2018). Mechanism and regulation of dynein motors In King S. M. (Ed.), Dyneins: Structure, biology and disease. Vol. 2: Dynein Mechanics, Dysfunction and Disease (pp. 37–51). Oxford, UK: Elsevier.

[cm21483-bib-0065] Schmidt, H. , Gleave, E. S. , & Carter, A. P. (2012). Insights into dynein motor domain function from a 3.3‐Å crystal structure. Nature Structural and Molecular Biology, 19, S491–S492. 10.1038/nsmb.2272 PMC3393637 22426545

[cm21483-bib-0066] Schmidt, H. , Zalyte, R. , Urnavicius, L. , & Carter, A. P. (2014). Structure of human cytoplasmic dynein‐2 primed for its power stroke. Nature, 518, 435. 25470043 10.1038/nature14023 PMC4336856

[cm21483-bib-0067] Shimizu, Y. , Sakakibara, H. , Kojima, H. , & Oiwa, K. (2014). Slow axonemal dynein e facilitates the motility of faster dynein c. Biophysical Journal, 106, 2157–2165. 24853744 10.1016/j.bpj.2014.04.009 PMC4052281

[cm21483-bib-0068] Shingyogi, C. (2018). Regulation of dynein‐driven ciliary and flagellar movement In King S. M. (Ed.), Dyneins: Structure, biology and disease. Volume 1 – The Biology of Dynein Motors (pp. 337–367). Oxford, UK: Elsevier.

[cm21483-bib-0069] Shingyoji, C. , Murakami, A. , & Takahashi, K. (1977). Local reactivation of triton‐extracted flagella by iontophoretic application of ATP. Nature, 265, 269–270. 834273 10.1038/265269a0

[cm21483-bib-0070] Smith, E. , & Yang, P. (2004). The radial spokes and central apparatus: Mechanochemical transducers that regulate flagellar motility. Cell Motility and the Cytoskeleton, 57, 8–17. 14648553 10.1002/cm.10155 PMC1950942

[cm21483-bib-0071] Steinman, J. B. , & Kapoor, T. (2018). Chemical probes for dynein In King S. M. (Ed.), Dyneins: Structure, biology and disease. Volume 2 – Dynein Mechanics, Dysfunction and Disease (pp. 173–191). Oxford, UK: Elsevier.

[cm21483-bib-0072] Summers, K. , & Gibbons, I. R. (1971). Adenosine triphosphate‐induced sliding of tubules in trypsin‐treated flagella of sea urchin sperm. Proceedings of the National Academy of Sciences of the United States of America, 68, 3092–3096. 5289252 10.1073/pnas.68.12.3092 PMC389597

[cm21483-bib-0073] Torisawa, T. , Ichikawa, M. , Furuta, A. , Saito, K. , Oiwa, K. , Kojima, H. , … Furuta, K. Y. (2014). Autoinhibition and cooperative activation mechanisms of cytoplasmic dynein. Nature Cell Biology, 16, 1118–1124. 25266423 10.1038/ncb3048

[cm21483-bib-0074] Toropova, K. , Mladenov, M. , & Roberts, A. J. (2017). Intraflagellar transport dynein is autoinhibited by trapping of its mechanical and track‐binding elements. Nature Structural and Molecular Biology, 24, 461–468. 10.1038/nsmb.3391 PMC5420313 28394326

[cm21483-bib-0075] Ueno, H. , Yasunaga, T. , Shingyoji, C. , & Hirose, K. (2008). Dynein pulls microtubules without rotating its stalk. Proceedings of the National Academy of Sciences of the United States of America, 105, 19702–19707. 19064920 10.1073/pnas.0808194105 PMC2604933

[cm21483-bib-0076] Wais‐Steider, J. , & Satir, P. (1979). Effect of vanadate on gill cilia: Switching mechanism in ciliary beat. Journal of Supramolecular Structure, 11, 339–347. 120905 10.1002/jss.400110309

[cm21483-bib-0077] Wickstead, B. (2018). The evolutionary biology of dyneins In King S. M. (Ed.), Dyneins: Structure, biology and disease. Volume 1 – The Biology of Dynein Motors (pp. 101–138). Oxford, UK: Elsevier.

[cm21483-bib-0078] Wickstead, B. , & Gull, K. (2007). Dyneins across eukaryotes: A comparative genomic analysis. Traffic, 8, 1708–1721. 17897317 10.1111/j.1600-0854.2007.00646.x PMC2239267

[cm21483-bib-0079] Wu, H. , Maciejewski, M. W. , Marintchev, A. , Benashski, S. E. , Mullen, G. P. , & King, S. M. (2000). Solution structure of a dynein motor domain associated light chain. Nature Structural Biology, 7, 575–579. 10876244 10.1038/76804

[cm21483-bib-0080] Yagi, T. , Minoura, I. , Fujiwara, A. , Saito, R. , Yasunaga, T. , Hirono, M. , & Kamiya, R. (2005). An axonemal dynein particularly important for flagellar movement at high viscosity: Implications from a new *Chlamydomonas* mutant deficient in the dynein heavy chain gene DHC9. Journal of Biological Chemistry, 280, 41412–41420. 16236707 10.1074/jbc.M509072200

[cm21483-bib-0081] Yamamoto, R. , Song, K. , Yanagisawa, H. , Fox, L. , Yagi, T. , Wirschell, M. , … Sale, W. S. (2013). The MIA complex is a conserved and novel dynein regulator essential for normal ciliary motility. Journal of Cell Biology, 201, 263–278. 23569216 10.1083/jcb.201211048 PMC3628515

[cm21483-bib-0082] Yamamoto, R. , Yanagisawa, H.‐A., Yagi, T., & Kamiya, R. (2008). Novel 44‐kilodalton subunit of axonemal dynein conserved from Chlamydomonas to mammals. Eukaryotic Cell, 7, 154–161. 17981992 10.1128/EC.00341-07 PMC2224156

[cm21483-bib-0083] Zhang, K. , Foster, H. E. , Rondelet, A. , Lacey, S. E. , Bahi‐Buisson, N. , Bird, A. W. , & Carter, A. P. (2017). Cryo‐EM reveals how human cytoplasmic dynein is auto‐inhibited and activated. Cell *,* 169, 1303–1314, e1318. 28602352 10.1016/j.cell.2017.05.025 PMC5473941

